# Physical realization of topological Roman surface by spin-induced ferroelectric polarization in cubic lattice

**DOI:** 10.1038/s41467-022-29764-w

**Published:** 2022-05-02

**Authors:** Guangxiu Liu, Maocai Pi, Long Zhou, Zhehong Liu, Xudong Shen, Xubin Ye, Shijun Qin, Xinrun Mi, Xue Chen, Lin Zhao, Bowen Zhou, Jia Guo, Xiaohui Yu, Yisheng Chai, Hongming Weng, Youwen Long

**Affiliations:** 1 Beijing National Laboratory for Condensed Matter Physics, Institute of Physics, Chinese Academy of Sciences, Beijing, China; 2 School of Physical Sciences, University of Chinese Academy of Sciences, Beijing, China; 3 Center of Quantum Materials and Devices, Chongqing University, Chongqing, China; 4 Low Temperature Physics Laboratory and Chongqing Key Laboratory of Soft Condensed Matter Physics and Smart Materials, College of Physics, Chongqing University, Chongqing, China; 5 Songshan Lake Materials Laboratory, Dongguan, Guangdong China

**Keywords:** Ferroelectrics and multiferroics, Magnetic properties and materials

## Abstract

Topology, an important branch of mathematics, is an ideal theoretical tool to describe topological states and phase transitions. Many topological concepts have found their physical entities in real or reciprocal spaces identified by topological invariants, which are usually defined on orientable surfaces, such as torus and sphere. It is natural to investigate the possible physical realization of more intriguing non-orientable surfaces. Herein, we show that the set of spin-induced ferroelectric polarizations in cubic perovskite oxides *A*Mn_3_Cr_4_O_12_ (*A* = La and Tb) reside on the topological Roman surface—a non-orientable two-dimensional manifold formed by sewing a Möbius strip edge to that of a disc. The induced polarization may travel in a loop along the non-orientable Möbius strip or orientable disc, depending on the spin evolution as controlled by an external magnetic field. Experimentally, the periodicity of polarization can be the same or twice that of the rotating magnetic field, which is consistent with the orientability of the disc and the Möbius strip, respectively. This path-dependent topological magnetoelectric effect presents a way to detect the global geometry of a surface and deepens our understanding of topology in both mathematics and physics.

## Abstract (web summary)

A non-orientable surface can mirror reflecting the man travelling on it. Realizing such topological object is fascinating. Here, the authors discover that antiferromagnetic-induced polarization in a solid can realize a non-orientable Roman surface.

## Introduction

Topological physics is one of the most successful integrations of the mathematics and physics field. As one of the branches of pure mathematics, topology has recently been employed to describe and understand many intriguing physical phenomena, such as the quantum Hall effect^1,2^, topological insulators, topological semimetals, and topological superconductors^3–5^. These topological electronic states are classified by various topological invariants, such as the Chern number *N*, topological classes *Z*_2_ and *Z*_4_, and Euler class *χ*^6–11^. It is known that the Chern number is defined as the integral of the vector field, i.e., the Berry curvature over the first Brillouin zone of a two-dimensional (2D) periodic system, which is considered as a torus (**T**^**2**^) and serves as the base manifold. Meanwhile, the magnetic spins and electric dipoles in real space can form topological objects or defects such as skyrmions^12,13^, polar skyrmions^14^, and polar merons^15^. These can be identified with an integer winding number^16^ based on the field of spin or electrical dipole orientations over the base manifold of a sphere (**S**^**2**^). In this sense, topological invariants are global characteristics, and topology-related physical properties are typically robust against perturbations^3,4,8^.

In addition to torus and sphere, there are three other fundamental 2D manifolds found in algebraic topology (see Supplementary Table 1): the Möbius strip, Klein bottle, and Roman surface. The torus and sphere are orientable and have two sides. The Berry curvature flux enclosed by them can be a nonzero quantized value and taken as a topological invariant, such as the Chern number. By contrast, the Möbius strip, Klein bottle, and Roman surface are non-orientable surfaces with only one side. The flux of any vector field through them is zero. A well-known example is that a one-sided Klein bottle cannot be filled with water, but a two-sided bottle can. Moreover, the non-orientable surfaces feature a geometrical property in which a body can be mirror-reflected when traveling along a special loop path on the surface, such as along the Möbius strip without crossing the boundary, and recovers after the second loop of travel. It will be intriguing to realize a physical system that can evolve globally on a non-orientable surface to reflect such unique geometrical properties.

In real space, a Möbius strip with a single boundary has been visualized in nanocrystal/DNA bands^17,18^. Möbius insulators have been proposed and realized in the reciprocal spaces of electronic^19–21^ and acoustic crystals^22,23^. Under proper nonsymmorphic symmetry or projective translation symmetry, the Bloch wavefunctions of its boundary states have 4$\pi$ periodicity instead of 2$\pi$, which mimics characteristics of a Möbius strip. Recently, nonorientability has been regarded as a *Z*_2_ gauge charge to describe the topological defect of shear deformation on a Möbius strip^24^. However, the smooth and global evolution of a physical quantity on a non-orientable surface and the detection of its intrinsic geometry remains a challenge. Furthermore, a non-orientable 2D surface without a boundary must self-intersect in a three-dimensional (3D) space, as shown by the Klein bottle^25^ (one-sided bottle) and Roman surface^26^ (**RP**^**2**^, real projective plane). This inhibits the physical realization and exhibition of topological behaviors in a 3D real space. Therefore, it is natural to ask whether the Roman surface, the simplest non-orientable 2D manifold without a boundary, can be realized by some physical entity and whether it can demonstrate exotic topological properties or phenomena.

**Fig. 1:**
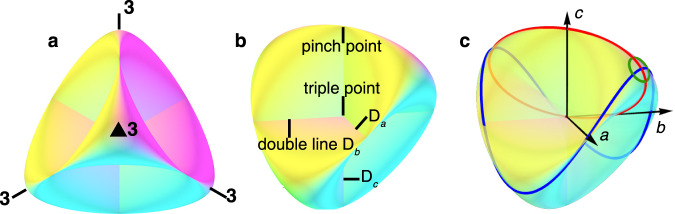
Schematic diagram of a Roman surface. **a**, **b** Roman surface with four three-fold axes (**3**), one triple point, six pinch points, and three double lines (D_*a*_, D_*b*_, D_*c*_). **c** Three geometrically different fundamental loops on a Roman surface. The red loop effectively winds twice to pass the two sides of the triple point as it rides on the Möbius strip being part of the Roman surface. The blue loop winds around the double line D_*c*_ once to have *W*_*c*_ = 1. The green loop does not enclose any double lines with zero *W*_*i*_ (*i* = *a*, *b*, *c*). Both blue and green loops only wind once to close the path.

Jakob Steiner discovered and named the Roman surface during his visit to Rome in early 1844. It is composed of a Möbius strip sewed to the edge of a disc and has a high tetrahedral T_d_ symmetry, which is different from other non-orientable 2D surfaces. Three self-intersecting double lines *D*_*i*_ (*i* = *a*, *b*, and *c* are three mutually perpendicular Cartesian axes) form a right trihedron in a Euclidean 3D phase space. Each double line ends with two pinch points, and they all intersect at the triple point (see Fig. 1 and Supplementary Fig. 1).

The triple point on the Roman surface indicates the intersection of the three surfaces. The analytical equation of the Roman surface **RP**^**2**^ in mathematics is

1 $${x}^{2}{y}^{2}+{x}^{2}{z}^{2}+{y}^{2}{z}^{2}-\delta xyz=0$$

where *δ* is a constant and (*x*, *y*, *z*) is a point in the *a*, *b*, and *c* coordinate systems. As a 2D manifold, there are three types of fundamental closed loops on a Roman surface, and other closed loops are formed by their combination. As Fig. 1c shows, the first type of loop is a red loop that passes through at least one point twice (e.g., this point can be the triple point). It winds twice to close the path on this non-orientable surface because it rides on the Möbius strip, which is a component of the Roman surface. The other two types of loops are blue and green. They are topologically different from the red ones because they do not pass any point twice and wind once to close the path. The blue loop can only wind along one of the double lines *D*_*i*_ (*i* = *a*, *b*, *c*) by *W*_*i*_ = 1 time. The green loop does not enclose any double lines and is topologically trivial. The physical entity that matches Eq. (1) may provide an opportunity to realize a Roman surface and exhibit path-dependent properties. Therefore, realizing the Roman surface is intriguing and may extend the territory in the field of topological physics.

Magnetoelectric (ME) multiferroics with spin-induced ferroelectric polarization (**P**) have received considerable attention owing to their coupled spin and electrical dipole orders^27^, where the electric polarization is switched using a magnetic field (**B**), and the magnetization (**M**) is switched using an electric field (**E**)^27,28^, giving rise to significant ME effects. A few physical mechanisms have been proposed for ME multiferroics^29–32^, providing a fertile and unique playground for investigating topological properties as a mapping between the spin vector space and electric dipole vector space. Although some structurally and ferroelectrically coupled vortex domain patterns have been observed in hexagonal *R*MnO_3_ (*R* = rare earth or Y, In) and BiFeO_3_^27,33^, spin-dependent topological multiferroics remain to be elucidated, the topological ME effects with versatile controls of **P** by **B** or **M** by **E** also need to be studied. Here, in *A*-site ordered cubic perovskites *A*Mn_3_Cr_4_O_12_ with high lattice symmetry, we discovered that the set of spin-induced ferroelectric polarizations can be described by a non-orientable Roman surface, as deduced through a 3D symmetry analysis. Various angle-dependent **P** oscillations under different magnetic fields were observed and can be understood based on the changes in the local topological structure and symmetry of the magnetism.

## Results

### Crystal structure of TbMn_3_Cr_4_O_12_

**Fig. 2:**
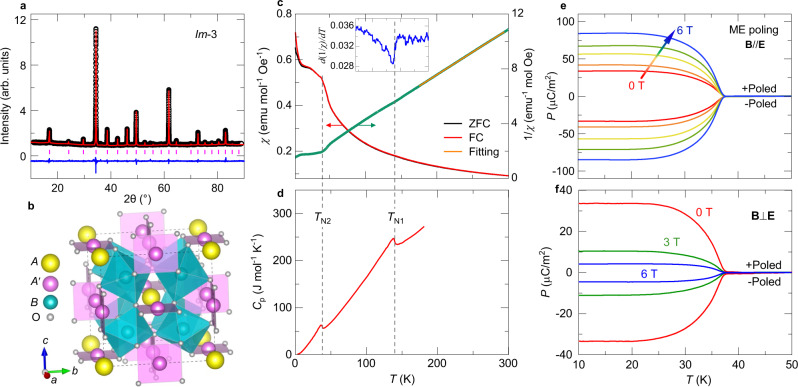
Structure characterization, magnetic, specific heat and polarization measurements for TMCO. **a** XRD pattern measured at room temperature and the Rietveld refinement results. The observed (black circles), calculated (red line), and difference (blue line) values are shown. The ticks indicate the allowed Bragg reflections with space group *Im*-3. **b** Crystal structure of *A*-site ordered perovskite *AA*′_3_*B*_4_O_12_ with *Im*-3 symmetry. **c** Temperature dependence of magnetic susceptibility *χ* measured at 0.1 T and its inverse *χ*^-1^. Both zero-field-cooling (ZFC) and field-cooling (FC) modes were adopted to measure *χ*. The yellow line shows the Curie–Weiss fitting above 175 K. The fitted effective moment *μ*_eff_ (15.52 *μ*_B_ f.u.^-1^) is comparable to the theoretical value (15.04 *μ*_B_ f.u.^-1^) by considering the contribution from Tb^3+^, Mn^3+^ and Cr^3+^ ions. The inset shows the derivative of inverse susceptibility versus temperature. **d** Temperature dependence of specific heat *C*_p_ measured at zero field. **e**, **f** Temperature dependent polarization *P* after poling the sample under an electric field (= **E**_pole_) and different magnetic fields **B**, where **B** was not removed until the temperature increased to 50 K. For **B**//**E** and **B**⊥**E** configurations, both +Poled and -Poled conditions were measured.

To prove the high cubic symmetry of synthesized TbMn_3_Cr_4_O_12_ (TMCO) at room temperature, we measured its powder X-ray diffraction (XRD) pattern and performed the Rietveld refinement (see Fig. 2a). The Rietveld analysis demonstrates that the compound crystallizes in an *A*-site ordered perovskite structure with the chemical formula *AA*′_3_*B*_4_O_12_ (see Fig. 2b)^34,35^. The space group is cubic *Im*-3 with central symmetry, where Tb and Mn are 1:3 ordered at special Wyckoff sites 2*a* (0, 0, 0) and 6*b* (0, 0.5, 0.5), and Cr and O at special 8*c* (0.25, 0.25, 0.25) and 24 *g* (*x*, *y*, 0) sites, respectively. Compared with a simple *AB*O_3_ perovskite, the introduction of a transition metal into the *A*′ site in the ordered *AA*′_3_*B*_4_O_12_ perovskite results in the formation of square-coordinated *A*′O_4_ units and heavily tilted *B*O_6_ octahedra, as shown in Fig. 2b and described in detail in literatures^36,37^. Supplementary Table 2 lists the refined structural parameters of TMCO. According to the refined Cr–O and Mn–O bond lengths, the bond valence sum calculations illustrate that the valence states of Mn and Cr are both close to +3, which agrees with the reported isostructural compound LaMn_3_Cr_4_O_12_ (LMCO)^38^. Moreover, when the temperature was decreased to 5 K, there is no trace of the structural phase transition in TMCO (see Supplementary Fig. 2).

### Magnetic properties of TbMn_3_Cr_4_O_12_

Figure 2c, d shows the temperature dependence of the magnetic susceptibility and specific heat of TMCO, respectively. With the temperature decreasing to *T*_N1_ ≈ 136 K, a sharp *λ*-type anomaly is observed in the specific heat, suggesting the occurrence of a second-order long-range magnetic phase transition. Moreover, the derivative of the inverse susceptibility displays a variation at *T*_N1_ (inset of Fig. 2c). Further, upon cooling to *T*_N2_ ≈ 36 K, both the susceptibility and specific heat show a clear anomaly, illustrating a second long-range magnetic transition. Below *T*_N2_, the specific heat does not show any visible anomaly, indicating the absence of spin ordering of Tb^3+^ at temperatures down to 2 K. The inverse susceptibility above 175 K follows the Curie–Weiss law (Fig. 2c). The fitted Weiss temperature of −28.0 K is indicative of antiferromagnetic (AFM) interactions. The linear isothermal magnetization behaviors within ±2 T further confirm the AFM orders occurring at *T*_N1_ and *T*_N2_ (see Supplementary Fig. 3a). However, at higher fields, a metamagnetic transition occurs near 2.5 T at temperatures below *T*_N1_ ≈ 136 K, as demonstrated by the field derivative of magnetization, indicating field-induced variation in spin alignment (Supplementary Fig. 3b). Similar to LMCO^38^, at zero field, the *B*-site Cr^3+^ spins are antiferromagnetically ordered in a collinear G-type fashion at *T*_N1_; the *A*′-site Mn^3+^ sublattice experiences another G-type AFM ordering at *T*_N2_ in the current TMCO. The total spin structure, composed of Cr^3+^ and Mn^3+^ magnetic sublattices with spin moments along the [111] direction, forms a type-II polar magnetic point group of 31′, which breaks the space inversion symmetry. Therefore, a spin-induced ferroelectric phase transition is expected to occur at *T*_N2_.

### Spin-induced ferroelectric polarization in TbMn_3_Cr_4_O_12_

Corresponding to the AFM phase transition, a frequency-independent dielectric peak is observed at *T*_N2_ ≈ 36 K in TMCO, whereas no dielectric anomaly emerges at *T*_N1_ (Supplementary Fig. 4a, b). When magnetic field **B** and electric field **E** are simultaneously applied to measure the dielectric constant, an apparent anisotropic behavior can be observed. Specifically, a sharp dielectric peak is always observed with **B** up to 9 T for **B**//**E** (Supplementary Fig. 4c). By contrast, **B** significantly suppresses the dielectric peak in the **B**⊥**E** configuration, whereas the dielectric anomaly nearly disappears at 6 T (Supplementary Fig. 4d). These results strongly suggest that spin-induced ferroelectric polarization also shows distinctive responses to external magnetic fields along the parallel and perpendicular configurations in TMCO^38^. Furthermore, when the pyroelectric current **I**_*p*_ was measured to obtain temperature-dependent polarization, a sharp ferroelectric phase transition was observed at *T*_N2_ (Fig. 2e), as expected from the formation of the polar magnetic point group mentioned earlier. The polarization is reversible without changing the magnitude if the sign of the poling electric field (**E**_pole_) is reversed, further confirming the spin-induced ferroelectric phase transition in TMCO. Similar to the dielectric measurements, applying a magnetic field with a **B**//**E** configuration can significantly enhance the polarization from 33.3 μC/m^2^ at 0 T to 84.4 μC/m^2^ at 6 T (Fig. 2e). However, in the **B**⊥**E** configuration, the spin-induced polarization is suppressed to 10.7 μC/m^2^ at **B** = 3 T, and only 4.51 μC/m^2^ at **B** = 6 T (Fig. 2f). All experimental results indicate that TMCO has the same crystal and magnetic structures as LMCO^38^.

### General relationship between spin and polarization directions of *A*Mn_3_Cr_4_O_12_

**Fig. 3:**
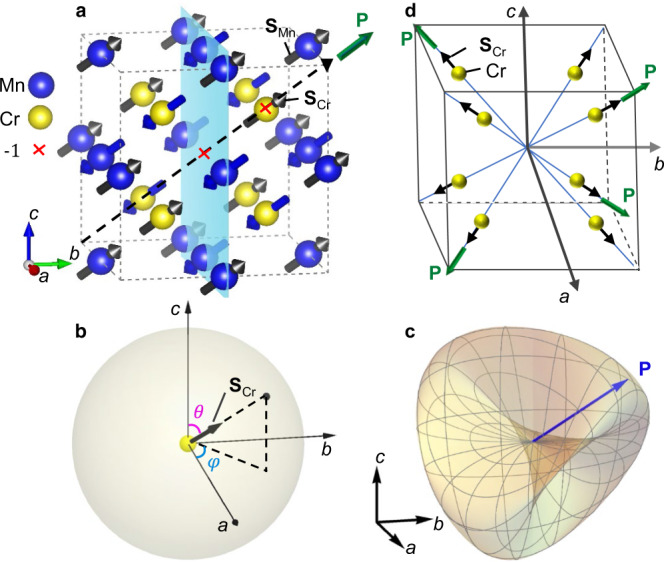
Crystal, magnetic, and ferroelectric structures in *A*Mn_3_Cr_4_O_12_. **a** Crystal and spin configurations where Cr and Mn atoms are presented in yellow and blue, respectively, and O atoms are omitted for clarity. The lattice symmetries of *A*Mn_3_Cr_4_O_12_: two space inversions $\bar{{{{{{\boldsymbol{1}}}}}}}$ located at the center and at the Cr atom, one of the mirror planes (see the light-blue plane), and one of the three-fold rotation axes (see the dash line) are indicated. All spins composed of Mn and Cr magnetic sublattices are parallel or antiparallel to the [111] direction. Antiferromagnetic sublattices of Cr and Mn are denoted by Néel vectors **S**_Cr_ and **S**_Mn_, respectively, and the induced polarization **P** is shown by the arrow in green. **b** Orientation of **S**_Cr_ is represented in spherical coordinates (*μ*_Cr_*, θ*, *φ*). **c** Complete set of allowed spin-induced **P** vectors in three dimensions is a Roman surface when the magnetic structure shown in **a** coherently rotates on a sphere in **b**. **d** Distribution of **P** vectors along four of the eight diagonal directions. The yellow sphere and the black arrow denote the Cr atom and its spin moment. **S**_Mn_ is always parallel to **S**_Cr,_ and it is not shown for clarity. Thus, AFM configuration composed of Mn and Cr sublattices is indicated by **S**_Cr_. The black cube represents the crystal unit cell.

Based on the crystal and spin structure symmetry analyses, the peculiar ME multiferroicity of *A*Mn_3_Cr_4_O_12_ can be described through the topological Roman surface **RP**^**2**^. As mentioned, both TMCO and LMCO have a cubic crystal symmetry of *Im*-3, where inversion centers are located on each Cr atom, with four three-fold rotation axes along four body diagonals and three mirror planes perpendicular to the three Cartesian axes (Fig. 3a). The total magnetic structure composed of Cr^3+^ spins (we use Néel vector **S**_Cr_ to trace the coherent motion of spins in the AFM ordering of Cr ions) and Mn^3+^ spins (Néel vector **S**_Mn_) forms a polar magnetic point group 31′ with **S**_Mn_//**S**_Cr_//[111] (see Fig. 3a). According to the proposed anisotropic symmetric exchange mechanism^39^ (see Supplementary Section 1) originating from spin–orbit coupling, such a special spin structure induces polarization **P** along the [111] direction. To describe a general relationship between the spin and polarization directions, we assign **S**_Cr_ = (*μ*_Cr_, *θ*, *φ*) and **S**_Mn_ = (*μ*_Mn_, *θ*, *φ*). Both are on sphere **S**^**2**^ (Fig. 3a and b), where $\theta \in \left[0,\pi \right]$ and $\varphi \in [-\pi ,\pi ]$ with fixed magnitudes *μ*_Cr_ and *μ*_Mn_. Thus, the polarization **P** is expressed as a function of spins **P**(**S**_Mn_, **S**_Cr_) = **P**(*θ*, *φ*) mapping from **S**^**2**^ to **RP**^**2**^. Based on strict symmetrical analysis^40^, as described in Supplementary Sections 2 and 3, we see that **P**(*θ*, *φ*) has the following form:

2 $${{{{{\bf{P}}}}}}={{{{{{\rm{\beta }}}}}}{{{{{\rm{\mu }}}}}}}_{{{{{{\rm{Cr}}}}}}}{\mu }_{{{{{{\rm{Mn}}}}}}}\Omega [\sin (2\theta )\sin (\varphi ),\,\sin (2\theta )\cos (\varphi ),\,{\sin }^{2}\theta \,\sin (2\varphi )]$$

where the coefficients β and Ω depend on temperature and crystal properties, respectively. The polarization vector **P**(*θ*, *φ*) = (*P*_*a*_, *P*_*b*_, *P*_*c*_) is rewritten using an analytical equation as follows:

3 $${P}_{a}^{2}{P}_{b}^{2}+{P}_{a}^{2}{P}_{c}^{2}+{P}_{b}^{2}{P}_{c}^{2}-2{{{{{{\rm{\beta }}}}}}{{{{{\rm{\mu }}}}}}}_{{{{{{\rm{Cr}}}}}}}{{{{{{\rm{\mu }}}}}}}_{{{{{{\rm{Mn}}}}}}}\Omega {P}_{a}{P}_{b}{P}_{c}=0$$

Equation (3) is a specific case of Eq. (1) with *x* = *P*_*a*_, *y* = *P*_*b*_, *z* = *P*_*c*_, and δ = 2βμ_Cr_*μ*_Mn_Ω. This result indicates that the induced **P** vectors in *A*Mn_3_Cr_4_O_12_ may constitute a non-orientable Roman surface **RP**^**2**^ (see Fig. 3c).

In this cubic multiferroic family, the collinear G-type AFM locks the Néel vectors **S**_Mn_ and **S**_Cr_ in parallel; they pass through all eight equivalent diagonal directions coherently (see Supplementary Section 3 for details). Only four of the eight diagonal directions are allowed for the polarization **P** vectors, which form a regular tetrahedron and agrees with the tetrahedral symmetry of the Roman surface (Fig. 3d). Note that there is another AFM configuration with **S**_Mn_ antiparallel to **S**_Cr_ (see Supplementary Fig. 5), which leads to a set of polarization vectors **P** forming a Roman surface with negative *δ* and having similar features as the positive one discussed here.

### Topological multiferroicity of *A*Mn_3_Cr_4_O_12_

The spin–polarization relationship in cubic perovskites *A*Mn_3_Cr_4_O_12_ leads to topological multiferroicity owing to the topological and symmetry properties of the Roman surface discussed earlier. The mapping from **S**^**2**^ to **RP**^**2**^ covers the Roman surface twice, because **S**_Cr_ and -**S**_Cr_ induce identical **P** vectors. This is similar to the spin–momentum locking effect on the Fermi surface enclosing Weyl nodes. The Hamiltonian around the Weyl node maps the momentum space (Fermi sphere, **S**^**2**^) to the spin space (another sphere called the Bloch sphere). If the enclosed Weyl node have a topological charge of one or two, the momentum moves around the node once and the spin rotates once or twice, respectively. The relative rotational directions of the momentum and spin are determined by the sign of the charge^41^. In this sense, a closed evolution loop of Néel vectors **S**_Cr_ on the sphere may map to a non-trivial path of the **P** vector on the Roman surface, showing topological ME effects.

**Fig. 4:**
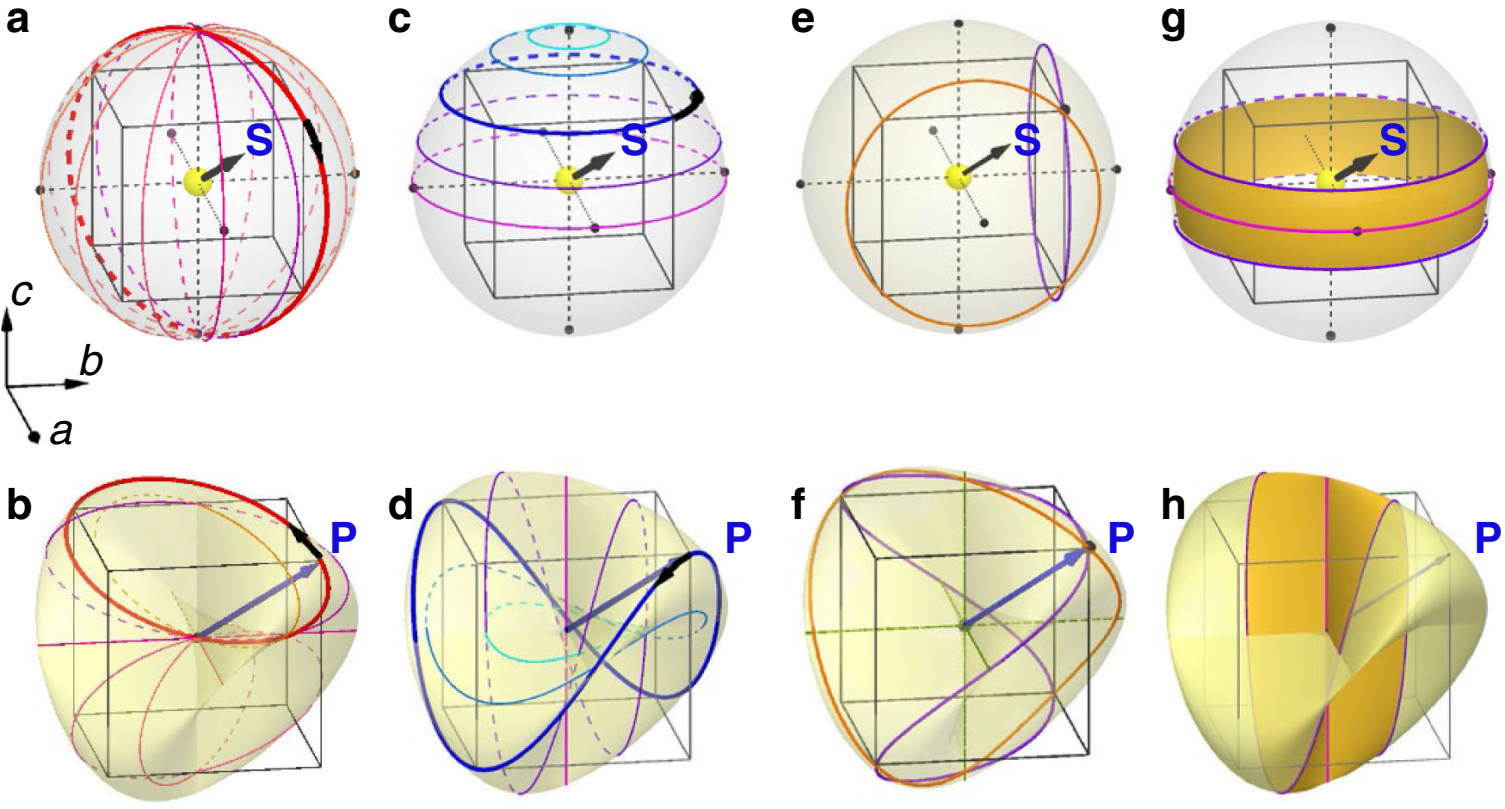
Various loops on a sphere of Néel vectors (top panels) and the induced fundamental polarization trajectories on Roman surface (bottom panels). **a** Longitudinal (fixed *φ*) path of the Néel vector rotation mode with a series of selected *φ* values and continuously varying $\theta \in \left[{{{{\mathrm{0,2}}}}}\pi \right]$. **b** Trajectories of **P** induced by loops in **a** with the same color. **c** Latitudinal (fixed *θ*) Néel vector rotation mode with a series of selected *θ* values and continuously varying $\varphi \in \left[-\pi ,\pi \right]$. **d** Trajectories of **P** induced by loops in **c** with the same color. The six special points, the intersections between sphere and *a*-, *b*- and *c*-axis, are illustrated in black point in **a** and **c**. The thicker lines in **a** and **c** represent two special cases where four diagonal directions are passed through; the resultant trajectories of **P** are indicated by the thicker lines in **b** and **d**, respectively. Rotation directions of **S**_Cr_ and **P** are indicated by black arrows toward opposite directions away from [111] direction. The animations of **a**–**d** are shown in Supplementary Videos 1–3. **e** Two closed loops winding the *a* and *b* axes once are shown in orange and purple, respectively. **f** Two closed trajectories of **P** on the Roman surface are induced by the loops in **e**. The loops with *W*_*a*_ = 1 are in orange and those with *W*_*b*_ = 1 are in purple. **g**, **h** A band included in the equator of the Néel vector sphere **g** is mapped to **h**, a self-crossed Möbius strip as a part of the Roman surface. The residual upper and lower caps are mapped to a disc on the Roman surface simultaneously. All the loops in **b**, **d**, and **f** are fundamental loops defined in Fig. 1c.

The red loop in Fig. 1c passes through the triple point of the Roman surface twice, where polarization **P** = 0. This can be induced by the evolution of **S**_Cr_ on sphere **S**^**2**^, which includes six special points where the sphere intersects the *a-, b-*, and *c-*axis (see Fig. 4a). For a longitudinal great circle of the sphere with *φ* fixed, as Fig. 4a shows, **S**_Cr_ passes through the special points twice (the north and south poles) if *φ* is not 0, π/2, π, or 3π/2. This results in a double-winding elliptical trajectory of the **P** vector passing through the triple point twice (see Fig. 4b and Supplementary Video 1). The induced **P** rotates twice, whereas **S**_Cr_ travels through one loop. When *φ* = 0, π/2, π, or 3π/2, the trajectories of **P** collapse to the double lines and pass through special points four times (see Fig. 4b and Supplementary Videos 1 and 2). It is observed that only the magnitude of **P** vibrates, and the period is twice the loop evolution of **S**_Cr_.

When the Néel vectors evolve along the latitudinal parallels with the polar axis being the *c*-axis and a fixed *θ* value (*θ* is not 0 or π/2), as Fig. 4c shows, the induced saddle-like trajectory of **P** on the Roman surface only winds around the *c*-axis (Fig. 4d and Supplementary Video 3). This trajectory of **P** is nontrivial, in the sense that *W*_*c*_ = 1. The projection onto the *ab*-plane is a circle. The rotation direction, clockwise or counterclockwise, of the projection of the **P** vector on the circle is opposite to that of the Néel vector. Note that the rotation period of the **P** vector is the same as that of **S**_Cr_, instead of double that in the cases shown in Fig. 4b. Similarly, when **S**_Cr_ winds around either the *a*- or *b*-axis once, the corresponding **P** vector loop on the Roman surface is characterized by *W*_*a*_ = 1 or *W*_*b*_ = 1, respectively, as indicated in Fig. 4e and f. Notably, nonzero *W*_*i*_ corresponds to the component of the **P** vector perpendicular to the *i*-axis that reverses *W*_*i*_ times during one loop evolution. The green circle in Fig. 1c is topologically trivial with zero values of *W*_*i*_. It can be mapped from a loop on the **S**_Cr_ sphere that satisfies these conditions: there is no special point being wound, and -**S**_Cr_ will not be passed by for any **S**_Cr_ on the path. To reveal the underlying topological origins of the double-winding and single-winding paths in Figs. 4b, d, and 1c, we mapped a strip composed of the neighborhoods of the equator and itself on the spin sphere (Fig. 4g) to a Möbius strip (Fig. 4h) on the Roman surface. Meanwhile, the residual upper and lower caps on the sphere are mapped to a saddle-like disc. Thus, according to the orientable and non-orientable nature of the disc and the Möbius strip, their paths on them will wind once and twice, respectively, to form a closed loop.

### Topological ME effects in *A*Mn_3_Cr_4_O_12_

**Fig. 5:**
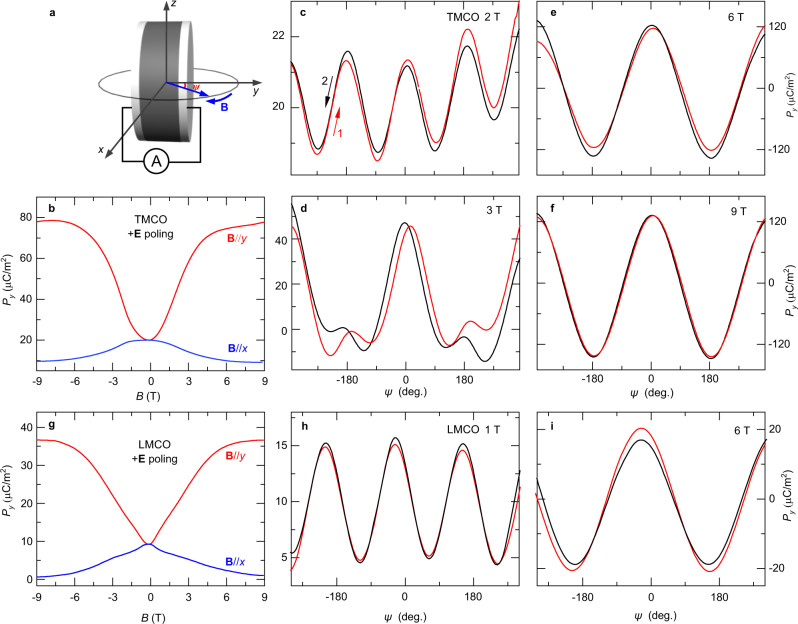
Polarization variations under sweeping and rotating B at 10 K for TMCO and LMCO. **a** Schematic illustration of measurements for magnetoelectric current under rotating **B** within the *xy-*plane; *ψ* denotes the angle between **B** and the *y*-direction. **b**, **g** Magnetic-field-dependent *P*_*y*_ at *T* = 10 K after +**E** poling for **B**//**E** and **B**⊥**E** configurations of TMCO and LMCO, respectively. *P*_*y*_ is defined as the component of **P** along the *y*-axis. **c**–**f** *ψ-*dependent polarization of TMCO at **B** = 2 T **c**, 3 T **d**, 6 T **e**, and 9 T **f**. The red and black arrows indicate an increase or decrease in angle, respectively. **h**, **i** *ψ-*dependent polarization of LMCO at **B** = 1 T **h** and 6 T **i**. All polarizations were integrated from the ME current as a function of time.

We experimentally present the topological ME effects in TMCO and LMCO polycrystalline samples with identical crystal and spin structures. To define the relative orientations of **E** and **B** on the polycrystalline sample, we defined a local *x*, *y*, and *z* coordinate system (see Fig. 5a). First, the samples were cooled down to 10 K under **E**_pole_//*y* at zero **B**. Subsequently, we removed the electric field **E**_pole_ and measured the **B***-*dependent **P** by sweeping **B** in either the **B**//*y* or **B**//*x* configuration (Fig. 5a). In both configurations, quadratic ME effects were dominant in TMCO (Fig. 5b). This is consistent with the 31′ point group with independent time-reversal symmetry, where a quadratic rather than a linear ME effect is allowed. To investigate the postulated topological ME effects in TMCO, rotating **B** was applied to continuously tune the orientation of the Néel vector **S**_Cr_. The **B** direction dependence of *P*_*y*_ under a selected field-strength by changing *ψ* (initially *ψ* = 0 with **B**//*y*; Fig. 5c–f) was measured after electrical poling to 10 K. The *ψ* dependence of *P*_*y*_ exhibited double-angle sinusoidal modulation curves at **B** within ~2 T without changing the sign (Fig. 5c). This is a direct manifestation of the intrinsic quadratic ME behavior in this system. Based on Monte Carlo simulations, we find that under a relatively small rotating **B** without inducing metamagnetic transition, the Néel vector **S**_Cr_ will slightly tilt away from the diagonal direction, leading to a topologically trivial path on the sphere. However, it is to be noted that **S**_Cr_ itself is double period of rotating **B** (see Supplementary Section 4 and Supplementary Fig. 6a). Moreover, the resulting loop of the **P** vector on the Roman surface is a small perturbation to its original position, with zero values of *W*_*i*_ (Supplementary Fig. 6b), as exemplified by the green loop in Fig. 1c. Therefore, **S**_Cr_ travels along a green-type path twice when **B** rotates 360° (Supplementary Fig. 7). This results in a double winding of the loop on the Roman surface, which is consistent with the experimentally observed double-angle sinusoidal modulation of *P*_*y*_ for small **B**, indicating trivial mapping from **S**_Cr_ to **P** along the green loop. Additionally, under applied magnetic field, **B** will change the orientation of antiferromagnetic axis and induce a small ferromagnetic component simultaneously. The Zeeman energy and antiferromagnetic exchange energy will compete with each other. The AFM components of spins tend to align normally with the direction of **B** while the induced ferromagnetic components point towards the **B** direction. This is also consistent with our Monte Carlo simulations under a small **B** in Supplementary Figs. 6a and 7a where **B**//*b* (*Φ* = 90°) leads to a significant weakening of ${S}_{{Cr}}^{y}$ in spins.

For **B** values greater than 3 T, a sinusoidal *P*_*y*_ variation appeared, owing to the metamagnetic transition occurring at a critical field of approximately 2.5 T (Supplementary Fig. 3b). At **B** = 6 and 9 T, the *P*_*y*_ value can be reversed with pure sinusoidal behavior (Fig. 5e, f). The energy barrier of magnetic anisotropy in the diagonal directions can be overcome by increasing the Zeeman energy, and a spin-flop transition occurs at a larger rotating **B** ( > 3 T) to induce a nontrivial loop of the Néel vector on the sphere. The loop of the Néel vector winds its principal axis under a sufficiently high **B**, which induces a **P** vector loop with one of *W*_*i*_ = 1 (*i* = *a*, *b*, *c*) on the Roman surface. This leads to a reversal of the **P** components perpendicular to the *i-*axis, as the blue loop in Fig. 1c. Thus, the pure sinusoidal **P** behavior at 6 T and 9 T in TMCO can be understood.

As Fig. 5d shows, both single- and double-angle sinusoidal *P*_*y*_ features coexist at **B** = 3 T with clear hysteresis behavior. *P* = 0 values have been reached multiple times in one period. There is a possibility that the loops of the Néel vector pass through some of the crystal axes, and some naught **P** values occur during a 360° rotation of **B**, as in the red loop in Fig. 1c. Note that owing to the polycrystalline specimens used in the experiments, it is impossible to determine the detailed grain configurations or **P** rotating paths of each grain for the TMCO. In isostructural LMCO, all the aforementioned ME behaviors can be reproduced (Fig. 5g–i), indicating the universality of these phenomena in the cubic multiferroic perovskite family of *A*Mn_3_Cr_4_O_12_.

## Discussion

We propose that topological nontrivial rotation of **P** is realized in the multiferroic domain walls of *A*Mn_3_Cr_4_O_12_ without an external magnetic field. As Supplementary Fig. 8 shows, there are three types of multiferroic domains with **S**_Mn_ parallel to **S**_Cr_, considering that the Néel vectors prefer eight diagonal directions, i.e., 71°, 109°, and 180°. AFM domains are induced at 109°, 109°, and 360°. In particular, ferroelectric domains are induced at 360°. The ferroelectric domain wall represents a half winding of the Möbius strip since a 180° rotation of **S**_Cr_ leads to one loop of **P** and a mirror reflected on the Roman surface (Supplementary Fig. 8c). This type of multiferroic domain wall cannot be destroyed by an external electric field because the polarizations in two neighboring ferroelectric domains can be coherently aligned in the direction of the electric field; whereas, the related AFM configurations will always be opposite. This would be a type of topological object unique in *A*Mn_3_Cr_4_O_12_, which deserves further investigation. Moreover, it is worthy to note that the magnetic skyrmion of Cu_2_OSeO_3_^42^ may also map the induced polarization as a complete Roman surface owing to the similar polarization distribution to that of *A*Mn_3_Cr_4_O_12_.

In summary, we investigated the unusual spin–polarization relationship in *A*-site-ordered cubic perovskite oxides *A*Mn_3_Cr_4_O_12_. The uncommon polarization occurring in the cubic system is coupled with the inversion symmetry breaking caused by G-type collinear AFM ordering composed of Mn^3+^ and Cr^3+^ magnetic sublattices. We discovered that the ME behavior, namely, the mapping from spin to the induced polarization, can be considered as a physical entity realizing the Roman surface, which is a non-orientable 2D manifold. This is different from other topological objects defined on orientable manifolds, such as Chern insulators defined on a torus and skyrmions defined on a sphere. The non-orientability of the Roman surface leads to topological ME effects depending on the evolution of the path. In our experiments, different types of angle-dependent polarization are observed in polycrystalline TMCO and LMCO under different magnetic fields. The evolution paths of the spin vectors are controlled by a magnetic field, and the induced **P** evolves along orientable or non-orientable surfaces to show different topological properties. This study provides an interesting fact that topology has a close and deep relationship with the physical world, opening up an avenue to achieve more topological objects from an orientable manifold to a non-orientable manifold.

## Methods

### Materials synthesis

Polycrystalline TMCO was synthesized via a solid-state reaction at a high temperature and high pressure. Tb_4_O_7_, MnO, Cr_2_O_3_, and Mn_2_O_3_ powders were used as the starting materials at a molar ratio of 1:2:8:5. The finely mixed reactants were packed into a gold capsule 4.0 mm in length and 2.8 mm in diameter and then treated at 1300 K and 7 GPa for 30 min on a cubic-anvil-type high-pressure apparatus.

### Structural determination

Powder XRD analysis was performed using a Huber diffractometer with Cu Kα1 radiation at 40 kV and 30 mA. Diffraction data were collected in the 2*θ* angle range of 10° to 100°. A program from the general structure analysis system was used to refine the XRD data using the Rietveld method^43^. Raman spectra were collected on a T64000 spectrometer using a liquid-nitrogen-cooled charge-coupled device. Volume Bragg gratings of 600 G and 488 nm laser were used to measure the spectra.

### Magnetic characterization

The magnetic susceptibility and magnetization were measured using a superconducting quantum interference device magnetometer (Quantum Design, MPMS-7T). Zero-field-cooling and field-cooling modes were adopted to measure the susceptibility at a magnetic field of 0.1 T. Magnetization was measured between −14 T and 14 T at selected temperatures. Specific heat measurements were performed using a physical property measurement system (Quantum Design, PPMS-9T) at zero field.

### Electric measurements

A hard disk-shaped piece 2.0 mm in diameter and 0.2 mm in thickness was used to measure the relative dielectric constant on an Agilent-4980A LCR meter. Silver paste was used as the electrodes. Subsequently, the sample was used to measure the pyroelectric current and isothermal polarized current under different magnetic fields using a Keithley 6517B electrometer. To measure the pyroelectric current for TMCO, the sample was poled with an electric field of **E**_pole_ =±  11.7 kV/cm and a magnetic field from 50 K to 10 K. Then, the electric field was removed. After waiting for 30 min in an electric short circuit, pyroelectric current data were collected at temperatures ranging from 10 K to 50 K at a speed of 2 K/min. Polarization *P* was obtained by integrating the current data as a function of time. The polycrystalline sample LMCO was also prepared in a disk-shaped piece with 0.23 mm in thickness to measure the polarization. In the measurements of magnetic-field dependent and angle-dependent polarizations, the polycrystalline sample was poled by an electric field of 8.33 kV/cm for TMCO and 8.68 kV/cm for LMCO down to 10 K. After the electrical poling procedure, different magnetic field was applied to the sample to measure the isothermal polarized current; then, the magnetic-field-dependent polarization or angle-dependent polarization was obtained by integrating the current.

### Reporting summary

Further information on research design is available in the Nature Research Reporting Summary linked to this article.

## Supplementary information

Peer Review File

Reporting Summary

Description of Additional Supplementary Files

Supplementary Video 1

Supplementary Video 2

Supplementary Video 3

Supplementary Informations

## Data Availability

The data supporting this study’s findings are available from the corresponding authors upon reasonable request.

## Code availability

The codes in this study are available from the corresponding authors upon reasonable request.
