# Supplementary material for: Physical realization of topological Roman surface by spin-induced ferroelectric polarization in cubic lattice
Source: Nat Commun. 2022 May 2;13:2373. doi: 10.1038/s41467-022-29764-w (PMC9061858; doi:10.1038/s41467-022-29764-w)
Supplement: Supplementary file 4 — Supplementary Video 1 [file 41467_2022_29764_MOESM4_ESM.pdf]

Description of Additional Supplementary Files

Supplementary Video 1

Supplementary Video 1 (mp4) displays the resultant trajectories of P vector on Roman surface due to longitudinal path of spin rotation mode with a series of selected φ values.

Supplementary Video 2

Supplementary Video 2 (mp4) displays the resultant trajectories of P vector on three double lines of Roman surface due to the spin rotation along three special mutually perpendicular great circles.

Supplementary Video 3

Supplementary Video 3 (mp4) displays the resultant trajectories of P vector on Roman surface due to latitudinal path of spin rotation mode with a series of selected θ values.
