# Supplementary material for: Physical realization of topological Roman surface by spin-induced ferroelectric polarization in cubic lattice
Source: Nat Commun. 2022 May 2;13:2373. doi: 10.1038/s41467-022-29764-w (PMC9061858; doi:10.1038/s41467-022-29764-w)
Supplement: Supplementary file 2 — Reporting Summary [file 41467_2022_29764_MOESM2_ESM.pdf]

nature portfolio

Peer Review File

Physical realization of topological Roman surface by spin-induced ferroelectric polarization in cubic lattice

Open Access This file is licensed under a Creative Commons Attribution 4.0 International License, which permits use, sharing, adaptation, distribution and reproduction in any medium or format, as long as you give appropriate credit to the original author(s) and the source, provide a link to the Creative Commons license, and indicate if changes were made. In the cases where the authors are anonymous, such as is the case for the reports of anonymous peer reviewers, author attribution should be to 'Anonymous Referee' followed by a clear attribution to the source work. The images or other third party material in this file are included in the article's Creative Commons license, unless indicated otherwise in a credit line to the material. If material is not included in the article's Creative Commons license and your intended use is not permitted by statutory regulation or exceeds the permitted use, you will need to obtain permission directly from the copyright holder. To view a copy of this license, visit http://creativecommons.org/licenses/by/4.0/.

REVIEWER COMMENTS

Reviewer #2 (Remarks to the Author):

I reviewed an earlier version of this manuscript and provided comments at that time, focusing mainly on the introduction and conclusion, which I felt were misleading. On the whole, I find that the authors have done a careful and effective job of modifying those sections to remove the suggestion that they have discovered a new topological phase of matter. The introduction now does a nice job of discussing at a rather high level the search for realizations in physics of topological constructions from mathematics, and setting the present work in that context.

My comments now are more concerned with the more technical discussion that starts around p. 7. I found it slightly disorienting that the manuscript jumped into these technical details so suddenly, but maybe it is unavoidable. If the authors can do something so smoothen this transition and introduce some of the experimental techniques in a way that is accessible to a broader audience, that would be to the good.

Around lines 189-192, am I supposed to understand something about what is going on? Like why E_parallel does one thing and E_perp does another? Or is it just a hint that there is some as-yet unspecified magnetoelectric behavior?

Line 216: it may be misleading to attribute P to a "ME coupling", since that phrase usually applies to a response to an external field.

Caption of Fig. 3: The discussion of symmetries (inversion, mirrors) is confusing because it is not properly explained that these are structural symmetries (defined by ignoring the magnetic order, which breaks these symmetries). The last line of the caption is also misleading: panel (a) shows a specific spin structure; what was meant was global rotations of the spins shown in (a), I guess.

My biggest confusion surrounds the fact that the experimental work is all on polycrystalline samples, whereas I would have thought that any direct confirmation of the predicted responses (double-period behavior etc.) would require single-crystal samples. I didn't understand well about the poling procedure; I think this has to be explained much more clearly. Here I refer to the text staring with line 318. What does poling under E_pole || y do? I guess that the domains that happen to have their local (111) axis roughly aligned to the y axis get poled, and other domains are left random. And in the following, the random domains act as bystanders, while the fraction of poled domains is responsible for the observed behavior. So, at this point, we can kind of imagine a single crystal with its (111) axis along y, except that

the magnitude of the response will be reduced by the smaller volume fraction and somewhat random orientation of the poled domains. Is this the idea?

OK, then E is removed and B is applied. What does B do? The material is an AFM, so nothing, to first order, I guess. Is it some higher-order effect? Does it favor parallel/antiparallel alignment of the spin direction with the field, or normal alignment? Why?

This all has to be spelled out and made clear to the non-expert reader.

The English is awkward in many places, and the text would benefit from polishing by a copy editor.

Response Letter to the Reviewer for NCOMMS-22-03425-T

First of all, we would like to thank the reviewer very much for his/her valuable comments and suggestions on our manuscript with the code NCOMMS-22-03425-T. According to the comments and suggestions, we revised the manuscript thoroughly. All the revisions are shown in red (see the highlighted PDF version for review). Our point-by-point response to the referee is shown as follows.

Response to Reviewer #2

Comment 1: I reviewed an earlier version of this manuscript and provided comments at that time, focusing mainly on the introduction and conclusion, which I felt were misleading. On the whole, I find that the authors have done a careful and effective job of modifying those sections to remove the suggestion that they have discovered a new topological phase of matter. The introduction now does a nice job of discussing at a rather high level the search for realizations in physics of topological constructions from mathematics, and setting the present work in that context.

Response 1: We thank the reviewer very much for his/her careful reading and these valuable comments and advices are very helpful. We have revised the manuscript shown in red according to the reviewer's suggestions.

Comment 2: My comments now are more concerned with the more technical discussion that starts around p. 7. I found it slightly disorienting that the manuscript jumped into these technical details so suddenly, but maybe it is unavoidable. If the authors can do something so smoothen this transition and introduce some of the experimental techniques in a way that is accessible to a broader audience, that would be to the good.

Response 2: Thanks for this comment. To smooth the transition, we have added some descriptions to emphasize the high symmetry of $A$$Mn_3Cr_4O_{12}$ as follows: "To prove the high cubic symmetry of the newly synthesized $TbMn_3Cr_4O_{12}$ (TMCO) at room

temperature, we measured its powder X-ray diffraction (XRD) pattern and performed the Rietveld refinement (see Fig. 2a).” For details, please see the red on page 7.

Comment 3: Around lines 189-192, am I supposed to understand something about what is going on? Like why E_parallel does one thing and E_perp does another? Or is it just a hint that there is some as-yet unspecified magnetoelectric behavior?

Response 3: It is indeed a hint of some unspecified magnetoelectric behaviors. Although the sample we measured is polycrystalline in nature, the dielectric constant shows different responses to *B* in different directions. It strongly suggests that “spin-induced ferroelectric polarization also shows distinctive responses to external magnetic fields along the parallel and perpendicular configurations in TMCO” (see the red on page 10). Actually, similar behaviors can also be found in polycrystalline LMCO [PRL, **115**, 087601, (2015)].

Comment 4: Line 216: it may be misleading to attribute P to a "ME coupling", since that phrase usually applies to a response to an external field.

Response 4: Thanks for this advice. As suggested by the reviewer, the “ME coupling” have been revised to “spin structure” shown in red on page 11 in the revised manuscript.

Comment 5: Caption of Fig. 3: The discussion of symmetries (inversion, mirrors) is confusing because it is not properly explained that these are structural symmetries (defined by ignoring the magnetic order, which breaks these symmetries). The last line of the caption is also misleading: panel (a) shows a specific spin structure; what was meant was global rotations of the spins shown in (a), I guess.

Response 5: As suggested by the reviewer, we have modified the caption of Fig.3a as: “The lattice symmetries of $AMn_3Cr_4O_{12}$: two space inversions $\bar{\mathbf{1}}$ located at the center…” to specify the structural symmetries only. The last line of the caption is also modified as: “Complete set of allowed spin-induced *P* vectors in three dimensions is a

Roman surface when the magnetic structure shown in **a** coherently rotates on a sphere in **c**."

Comment 6: My biggest confusion surrounds the fact that the experimental work is all on polycrystalline samples, whereas I would have thought that any direct confirmation of the predicted responses (double-period behavior etc.) would require single-crystal samples. I didn't understand well about the poling procedure; I think this has to be explained much more clearly. Here I refer to the text staring with line 318. What does poling under E_{pole} || y do? I guess that the domains that happen to have their local (111) axis roughly aligned to the y axis get poled, and other domains are left random. And in the following, the random domains act as bystanders, while the fraction of poled domains is responsible for the observed behavior. So, at this point, we can kind of imagine a single crystal with its (111) axis along y, except that the magnitude of the response will be reduced by the smaller volume fraction and somewhat random orientation of the poled domains. Is this the idea?

Response 6: Thanks for these comments. We agree with the referee that a definite direct confirmation of the predicted responses (double-period behavior etc.) would require single-crystal samples. We are still doing our best to grow the single crystal, but this mission is very tough due to the extremely high pressure. Anyway, we hope the current results based on polycrystalline can provide useful information. As commented by the referee, to clarify the poling procedure more clearly, some descriptions have been added as "To determine the relative orientations of *E* and *B* on polycrystalline sample, we defined a local *x*, *y*, and *z* coordinate system (see Fig. 5a)" (see the red on page 16). About the poling under E_{pole} || y, the main idea proposed by the reviewer is correct. Strictly speaking, as a polycrystalline ferroelectric sample with cubic symmetry and diagonal-direction polarization, the effect of poling electric field is very similar to that of a magnetic field on a polycrystalline ferromagnetic sample with cubic symmetry and diagonal-direction easy axis. In both cases, the ferroelectric/ferromagnetic domains will be aligned within $cos^{-1}\frac{1}{\sqrt{3}} \approx 55°$ spherical

angle to the y axis. And in the following, the poled domains with the preferred orientation are responsible for the observed behaviors. So, at this point, one can kind of imagine a single crystal with its (111) axis along y and the net P will be reduced by the slightly deviation of (111) axis of different domains.

Comment 7: OK, then E is removed and B is applied. What does B do? The material is an AFM, so nothing, to first order, I guess. Is it some higher-order effect? Does it favor parallel/antiparallel alignment of the spin direction with the field, or normal alignment? Why? This all has to be spelled out and made clear to the non-expert reader.

Response 7: Under applied magnetic field, B will change the orientation of antiferromagnetic axis and induce a small ferromagnetic component simultaneously. The B induced M is rather weak in experiments [PRL **115**, 087601 (2015) for $LaMn_3Cr_4O_{12}$] so that it may be a higher-order effect. Meanwhile, the Zeeman energy and antiferromagnetic exchange energy will compete with each other. The AFM components of spins tend to align normally with the direction of B while the induced ferromagnetic components point towards the B direction, as shown in Fig. R1 shown below. This is also consistent with our Monte Carlo simulations under a small B in Supplementary Figs. 6a and 7a where $B//b$ (Φ = 90°) leads to a significant weakening of S_{Cr}^{y} in spins. As suggested by the reviewer, we have added detailed descriptions in the revised manuscript as shown in red on page 18.

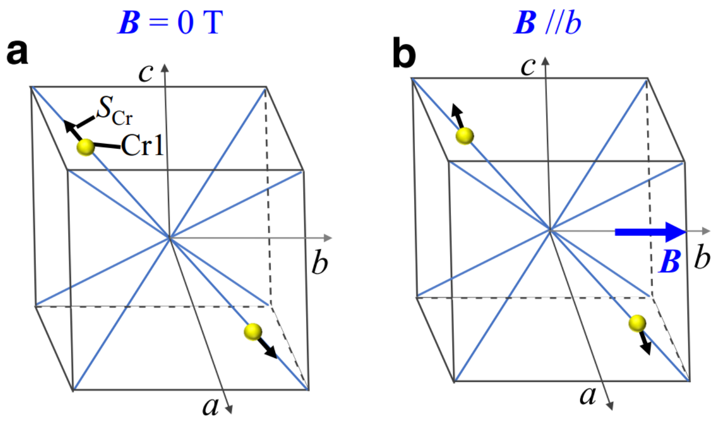

Fig. R1 | Schematic illustration of variation of AFM configurations represented by S_{Cr} under applied *B*. **a**,**b** Two AFM configurations under zero *B* (**a**) and applied *B* toward *b*-axis (**b**).

Comment 8: The English is awkward in many places, and the text would benefit from polishing by a copy editor.

Response 8: As suggested by the referee, the English have been edited and polished carefully by a specialized company (the Editage).

REVIEWERS' COMMENTS

Reviewer #2 (Remarks to the Author):

For the most part, the authors have done a reasonable job of taking my previous referee comments into account. I still find the second half of the paper to be somewhat technical and difficult to follow, especially in view of the unfortunate unavailability of single-crystal samples. However, overall I think the paper makes a nice point about how the symmetry of the magnetoelectric response allows a nontrivial topological dependence of electric polarization on magnetic field, and confirms it with convincing experimental evidence. As a result, I am prepared to recommend it for publication.

Response Letter to the Reviewer for NCOMMS-22-03425A

Response to Reviewer #2

Comment: For the most part, the authors have done a reasonable job of taking my previous referee comments into account. I still find the second half of the paper to be somewhat technical and difficult to follow, especially in view of the unfortunate unavailability of single-crystal samples. However, overall I think the paper makes a nice point about how the symmetry of the magnetoelectric response allows a nontrivial topological dependence of electric polarization on magnetic field, and confirms it with convincing experimental evidence. As a result, I am prepared to recommend it for publication.

Response: We appreciate the reviewer very much for the positive comments as well as the recommendation for publication in Nature Communications. Stimulated by the referee's valuable comments, our manuscript has been improved extensively.
