# Supplementary material for: Physical realization of topological Roman surface by spin-induced ferroelectric polarization in cubic lattice
Source: Nat Commun. 2022 May 2;13:2373. doi: 10.1038/s41467-022-29764-w (PMC9061858; doi:10.1038/s41467-022-29764-w)
Supplement: Supplementary file 1 — Peer Review File [file 41467_2022_29764_MOESM1_ESM.pdf]

Supplementary Information for

Physical realization of topological Roman surface by spin-induced ferroelectric polarization in cubic lattice

Guangxiu Liu, Maocai Pi, Long Zhou, Zhehong Liu, Xudong Shen, Xubin Ye, Shijun Qin, Xinrun Mi, Xue Chen, Lin Zhao, Bowen Zhou, Jia Guo, Xiaohui Yu, Yisheng Chai*, Hongming Weng*, Youwen Long*

*Corresponding author E-mail: yschai@cqu.edu.cn (Y.C.); hmweng@iphy.ac.cn (H.W.); ywlong@iphy.ac.cn (Y.L.)

Content

1. Microscopic origin of polarization in cubic AMn$_3$Cr$_4$O$_{12}$ (A = La, Tb)
2. Derive of the matrix form of two spin tensors
3. Polarization calculation
4. The Monte Carlo simulations under a small rotating B
5. References

1. Microscopic origin of polarization in cubic $AMn_3Cr_4O_{12}$

The most three well-known microscopic origin mechanisms of spin induced polarization (**P**) can be written as[1,2]:

$$\mathbf{P} = P^{MS}(\mathbf{S}_1 \cdot \mathbf{S}_2)\mathbf{e}_{12} + P^{SP}\mathbf{e}_{12}\times(\mathbf{S}_1\times\mathbf{S}_2) + P^{ORB}[\mathbf{e}_1(\mathbf{e}_1 \cdot \mathbf{S}_1)^2 - \mathbf{e}_2(\mathbf{e}_2 \cdot \mathbf{S}_2)^2] \quad \text{(S1)}$$

where the spins $\mathbf{S}_1$ and $\mathbf{S}_2$ reside on the adjacent sites along the unit vector $\mathbf{e}_1$, $\mathbf{e}_2$. The $\mathbf{e}_1$ and $\mathbf{e}_2$ are unit vectors pointing from site *1* and site *2* to ligand atom, respectively. P^{MS}, P^{SP} and P^{ORB} are scalars determined by the exchange interaction, the Dzyaloshinskii-Moriya (DM) interaction and *p-d* hybridization, respectively. All the above three mechanisms can be expressed in two spin tensor in matrix form as:

$$P^{\alpha\beta\gamma}_{exchange} = P^{MS}\begin{pmatrix} e_x, e_y, e_z & 0,0,0 & 0,0,0 \\ 0,0,0 & e_x, e_y, e_z & 0,0,0 \\ 0,0,0 & 0,0,0 & e_x, e_y, e_z \end{pmatrix} \quad \text{(S2)}$$

$$P^{\alpha\beta\gamma}_{DM} = P^{SP}\begin{pmatrix} 0,0,0 & e_y, -e_x, 0 & e_z, 0, -e_x \\ -e_y, e_x, 0 & 0,0,0 & 0, e_z, -e_y \\ -e_z, 0, e_x & 0, -e_z, e_y & 0,0,0 \end{pmatrix} \quad \text{(S3)}$$

$$P^{\alpha\beta\gamma}_{hybridization} = P^{ORB}\begin{pmatrix} e_xe_xe_x, e_xe_xe_y, e_xe_xe_z & e_xe_ye_x, e_xe_ye_y, e_xe_ye_z & e_xe_ze_x, e_xe_ze_y, e_xe_ze_z \\ e_ye_xe_x, e_ye_xe_y, e_ye_xe_z & e_ye_ye_x, e_ye_ye_y, e_ye_ye_z & e_ye_ze_x, e_ye_ze_y, e_ye_ze_z \\ e_ze_xe_x, e_ze_xe_y, e_ze_xe_z & e_ze_ye_x, e_ze_ye_y, e_ze_ye_z & e_ze_ze_x, e_ze_ze_y, e_ze_ze_z \end{pmatrix} \quad \text{(S4)}$$

From the polarization expressions of Eqs. S7 and S15, we can see that the effective components $P^{a,b,c}+P^{b,c,a}+P^{c,a,b}+P^{a,c,b}+P^{b,a,c}+P^{c,b,a}$ in $P^{\alpha\beta\gamma}_{ij}$ do not match with any known microscopic mechanism in Eqs. S2-S4. Therefore, the intrinsic polarization of $TbMn_3Cr_4O_{12}$ TMCO is governed by a new mechanism, anisotropic symmetric exchange mechanism due to spin orbital coupling[3].

2. Derive of the matrix form of two spin tensors

In general, the third-order local ME two-spin tensor $P_{12}^{\alpha\beta\gamma}$ of Cr-Mn bond $\langle 1, 2\rangle$ in Supplementary Fig. 5a can be expressed as in the matrix form[4]**:**

$$P_{12}^{\alpha\beta\gamma} = \begin{pmatrix} \{P^{a,a,a}, P^{a,a,b}, P^{a,a,c}\} & \{P^{a,b,a}, P^{a,b,b}, P^{a,b,c}\} & \{P^{a,c,a}, P^{a,c,b}, P^{a,c,c}\} \\ \{P^{b,a,a}, P^{b,a,b}, P^{b,a,c}\} & \{P^{b,b,a}, P^{b,b,b}, P^{b,b,c}\} & \{P^{b,c,a}, P^{b,c,b}, P^{b,c,c}\} \\ \{P^{c,a,a}, P^{c,a,b}, P^{c,a,c}\} & \{P^{c,b,a}, P^{c,b,b}, P^{c,b,c}\} & \{P^{c,c,a}, P^{c,c,b}, P^{c,c,c}\} \end{pmatrix} \quad \text{(S5)}$$

After considering the five symmetry operations (3-fold along [1,1,1], space inversion at each Cr and three mirror operations perpendicular to *a*, *b*, *c*-axes), all the 48 Cr-Mn bonds related two-spin tensor $P_{ij}^{\alpha\beta\gamma}$ can be deduced from the original $P_{12}^{\alpha\beta\gamma}$.

$P_{ij}^{\alpha\beta\gamma}$ is a polar tensor. Therefore, the general transformation rule for a polar tensor of order 3 is $T'^{ijk} = a_{il}a_{jm}a_{kn}T^{lmn}$, of which a_{il}, a_{jm} and a_{kn} are direction cosines (or transformation operators), T'^{ijk} and T^{lmn} are tensor component in new coordinate system and old coordinate system, respectively.

Then we present the transformations of $P_{12}^{\alpha\beta\gamma}$ under five specific symmetry operations in TMCO lattice:

1) The direction cosines of 3-fold rotation along [1,1,1] can be expressed as:

$$\mathbf{3}_{[111]} = \begin{pmatrix} 0 & 1 & 0 \\ 0 & 0 & 1 \\ 1 & 0 & 0 \end{pmatrix} \quad \text{(S6)}$$

Then the transformed $P'^{\alpha\beta\gamma}_{ij}$ can be expressed as (if bond $\langle 1, 2\rangle$ will be transformed to bond $\langle i, j\rangle$):

$$P'^{\alpha\beta\gamma}_{ij} = \begin{pmatrix} \{P^{c,c,c}, P^{c,c,a}, P^{c,c,b}\} & \{P^{c,a,c}, P^{c,a,a}, P^{c,a,b}\} & \{P^{c,b,c}, P^{c,b,a}, P^{c,b,b}\} \\ \{P^{a,c,c}, P^{a,c,a}, P^{a,c,b}\} & \{P^{a,a,c}, P^{a,a,a}, P^{a,a,b}\} & \{P^{a,b,c}, P^{a,b,a}, P^{a,b,b}\} \\ \{P^{b,c,c}, P^{b,c,a}, P^{b,c,b}\} & \{P^{b,a,c}, P^{b,a,a}, P^{b,a,b}\} & \{P^{b,b,c}, P^{b,b,a}, P^{b,b,b}\} \end{pmatrix} \quad \text{(S7)}$$

2) The direction cosines of $\bar{\mathbf{1}}$ can be expressed as:

$$\bar{\mathbf{1}} = \begin{pmatrix} -1 & 0 & 0 \\ 0 & -1 & 0 \\ 0 & 0 & -1 \end{pmatrix} \quad \text{(S8)}$$

Then the transformed $P'^{\alpha\beta\gamma}_{kl}$ can be expressed as (if bond $\langle 1, 2\rangle$ will be transformed to bond $\langle k, l\rangle$):

$$P'^{\alpha\beta\gamma}_{kl} = \begin{pmatrix} \{-P^{a,a,a}, -P^{a,a,b}, -P^{a,a,c}\} & \{-P^{a,b,a}, -P^{a,b,b}, -P^{a,b,c}\} & \{-P^{a,c,a}, -P^{a,c,b}, -P^{a,c,c}\} \\ \{-P^{b,a,a}, -P^{b,a,b}, -P^{b,a,c}\} & \{-P^{b,b,a}, -P^{b,b,b}, -P^{b,b,c}\} & \{-P^{b,c,a}, -P^{b,c,b}, -P^{b,c,c}\} \\ \{-P^{c,a,a}, -P^{c,a,b}, -P^{c,a,c}\} & \{-P^{c,b,a}, -P^{c,b,b}, -P^{c,b,c}\} & \{-P^{c,c,a}, -P^{c,c,b}, -P^{c,c,c}\} \end{pmatrix} \quad \text{(S9)}$$

3) The direction cosines of mirrors perpendicular to *a*-axis, *b*-axis and *c*-axis can be expressed as:

$$\boldsymbol{m}_a = \begin{pmatrix} -1 & 0 & 0 \\ 0 & 1 & 0 \\ 0 & 0 & 1 \end{pmatrix} \; \boldsymbol{m}_b = \begin{pmatrix} 1 & 0 & 0 \\ 0 & -1 & 0 \\ 0 & 0 & 1 \end{pmatrix} \; \boldsymbol{m}_c = \begin{pmatrix} 1 & 0 & 0 \\ 0 & 1 & 0 \\ 0 & 0 & -1 \end{pmatrix}, \text{ respectively} \quad \text{(S10)}$$

Then the transformed $P'^{\alpha\beta\gamma}_{mn}$, $P'^{\alpha\beta\gamma}_{op}$, $P'^{\alpha\beta\gamma}_{qr}$ can be expressed as (if bond $\langle 1, 2\rangle$ will be transformed to bond $\langle m, n\rangle$, $\langle o, p\rangle$,$\langle q, r\rangle$, respectively):

$$P'^{\alpha\beta\gamma}_{mn} = \begin{pmatrix} \{-P^{a,a,a}, P^{a,a,b}, P^{a,a,c}\} & \{P^{a,b,a}, -P^{a,b,b}, -P^{a,b,c}\} & \{P^{a,c,a}, -P^{a,c,b}, -P^{a,c,c}\} \\ \{P^{b,a,a}, -P^{b,a,b}, -P^{b,a,c}\} & \{-P^{b,b,a}, P^{b,b,b}, P^{b,b,c}\} & \{-P^{b,c,a}, P^{b,c,b}, P^{b,c,c}\} \\ \{P^{c,a,a}, -P^{c,a,b}, -P^{c,a,c}\} & \{-P^{c,b,a}, P^{c,b,b}, P^{c,b,c}\} & \{-P^{c,c,a}, P^{c,c,b}, P^{c,c,c}\} \end{pmatrix} \quad \text{(S11)}$$

$$P'^{\alpha\beta\gamma}_{op} = \begin{pmatrix} \{P^{a,a,a}, -P^{a,a,b}, P^{a,a,c}\} & \{-P^{a,b,a}, P^{a,b,b}, -P^{a,b,c}\} & \{P^{a,c,a}, -P^{a,c,b}, P^{a,c,c}\} \\ \{-P^{b,a,a}, P^{b,a,b}, -P^{b,a,c}\} & \{P^{b,b,a}, -P^{b,b,b}, P^{b,b,c}\} & \{-P^{b,c,a}, P^{b,c,b}, -P^{b,c,c}\} \\ \{P^{c,a,a}, -P^{c,a,b}, P^{c,a,c}\} & \{-P^{c,b,a}, P^{c,b,b}, -P^{c,b,c}\} & \{P^{c,c,a}, -P^{c,c,b}, P^{c,c,c}\} \end{pmatrix} \quad \text{(S12)}$$

$$P'^{\alpha\beta\gamma}_{qr} = \begin{pmatrix} \{P^{a,a,a}, P^{a,a,b}, -P^{a,a,c}\} & \{P^{a,b,a}, P^{a,b,b}, -P^{a,b,c}\} & \{-P^{a,c,a}, -P^{a,c,b}, P^{a,c,c}\} \\ \{P^{b,a,a}, P^{b,a,b}, -P^{b,a,c}\} & \{P^{b,b,a}, P^{b,b,b}, -P^{b,b,c}\} & \{-P^{b,c,a}, -P^{b,c,b}, P^{b,c,c}\} \\ \{-P^{c,a,a}, -P^{c,a,b}, P^{c,a,c}\} & \{-P^{c,b,a}, -P^{c,b,b}, P^{c,b,c}\} & \{P^{c,c,a}, P^{c,c,b}, -P^{c,c,c}\} \end{pmatrix} \quad \text{(S13)}$$

All other all Cr-Mn bonds related two-spin tensor $P^{\alpha\beta\gamma}_{ij}$ can be deduced by combining these above symmetry operations.

3. Polarization calculation

In these cubic *A*-site ordered perovskite systems with collinear *G*-type antiferromagnetic (AFM) alignments, there are two kinds of AFM configurations connected by space inversion operation at the center of unit cell, as schematically shown in Fig. 3a and Supplementary Fig. 5a.[3] In Supplementary Fig. 5a, the Néel vectors $\mathbf{S}_{\mathrm{Mn}}$ and $\mathbf{S}_{\mathrm{Cr}}$ are always antiparallel with each other. The corresponding **P** vector is opposite between the two AFM configurations when all the spins are aligned along the same diagonal direction. From the symmetry analysis, when magnetic structure points to any diagonal direction, a ferroelectric polarization **P** along the same diagonal axis will be induced in both spin configurations. When $\mathbf{S}_{\mathrm{Mn}}$ and $\mathbf{S}_{\mathrm{Cr}}$ coherently go over all eight equivalent directions, there are eight possible orientations of **P** vector along [±1,±1,±1].

By assigning AFM configuration of $\mathbf{S}_{\mathrm{Cr}}$ and $\mathbf{S}_{\mathrm{Mn}}$ at each site, we can calculate the total polarization $\boldsymbol{P}(\mathbf{S}_{\mathrm{Mn}}, \mathbf{S}_{\mathrm{Cr}})$ of one magnetic unit cell via

$$P^{\gamma}(\mathbf{S}_{\mathrm{Mn}}, \mathbf{S}_{\mathrm{Cr}}) = \sum_{<i,j>} P_{ij}^{\alpha\beta\gamma} S_{\mathrm{Mn}\alpha} S_{\mathrm{Cr}\beta}, \ (\alpha, \beta = a, b, c), \qquad \text{(S14)}$$

First we assume Néel vector $\mathbf{S}_{\mathrm{Cr}} = (1/\sqrt{3})\mu_{\mathrm{Cr}}[\pm1,\pm1,\pm1]$ and $\mathbf{S}_{\mathrm{Mn}} = (1/\sqrt{3})\mu_{\mathrm{Mn}}[\pm1,\pm1,\pm1]$ along [±1,±1,±1] directions at zero magnetic field. Substituting $\mathbf{S}_{\mathrm{Cr}}$ and $\mathbf{S}_{\mathrm{Mn}}$ and Eqs. S7, S9, S11 to S13 into Eq. S14 and summing over one magnetic unit cell of AFM phase, the $\mathbf{P}(\mathbf{S}_{\mathrm{Mn}}, \mathbf{S}_{\mathrm{Cr}})$ can calculated as:

$$\boldsymbol{P}(\mathbf{S}_{\mathrm{Mn}}, \mathbf{S}_{\mathrm{Cr}}) \propto (16/3)\, \mu_{\mathrm{Mn}}\mu_{\mathrm{Cr}}(P^{a,b,c}+P^{b,c,a}+P^{c,a,b}+P^{a,c,b}+P^{b,a,c}+P^{c,b,a})\, [\pm1,\pm1,\pm1] \qquad \text{(S15)}$$

From the expression we know that only six components in $P_{12}^{\alpha\beta\gamma}$ contribute to net polarization when $\mathbf{S}_{\mathrm{Mn}}//\mathbf{S}_{\mathrm{Cr}}//$ [±1,±1,±1]. For simplicity, we let

$$\Omega = P^{a,b,c} + P^{b,c,a} + P^{c,a,b} + P^{a,c,b} + P^{b,a,c} + P^{c,b,a} \quad \text{(S16)}$$

leading to a simplified Eq. S15 as:

$$\mathbf{P}(\mathbf{S}_{\mathrm{Mn}}, \mathbf{S}_{\mathrm{Cr}}) \propto (16/3)\, \mu_{\mathrm{Mn}}\mu_{\mathrm{Cr}}\Omega[\pm1,\pm1,\pm1] \quad \text{(S17)}$$

As for the magnetic structure shown in Fig. 3a is concerned, [±1,±1,±1] comprises only combinations with even numbers of the minus sign. Therefore, there are only four among eight diagonal directions are allowed for the polarization vectors. These allowed **P** vectors form a regular tetrahedron (Fig. 3d). For the spin configuration of Supplementary Fig. 5a, spin-induced **P** vector can only occupy the rest four directions forming the other tetrahedron (Supplementary Fig. 5b). Along any diagonal directions, the two spin configurations generate opposite **P** vectors.

To further reveal the spin-polarization relationship when $\mathbf{S}_{\mathrm{Cr}}$ points to an arbitrary orientation in a 3D space, we assumed $\mathbf{S}_{\mathrm{Cr}} = (\mu_{\mathrm{Cr}}, \theta, \varphi)$ and $\mathbf{S}_{\mathrm{Mn}} = (\mu_{\mathrm{Mn}}, \theta, \varphi)$ (Fig. 3a and b) in a spherical coordinate with $\theta = (0, \pi)$ and $\varphi = (-\pi, \pi)$. By substituting $\mathbf{S}_{\mathrm{Cr}}$ and $\mathbf{S}_{\mathrm{Mn}}$ into Eq. S14 and summing over one magnetic unit cell of the AFM phase, the $\mathbf{P}(\mathbf{S}_{\mathrm{Mn}}, \mathbf{S}_{\mathrm{Cr}})$ can be converted into a parameter equation $\mathbf{P}(\theta, \varphi)$ as follows:

$$\mathbf{P} = \beta\mu_{\mathrm{Cr}}\mu_{\mathrm{Mn}}\Omega[\sin(2\theta)\sin(\varphi), \sin(2\theta)\cos(\varphi), \sin^2\theta\sin(2\varphi)], \quad \text{(S18)}$$

where the coefficient β depends on the temperature and magnetic field. The analytical equation of Eq. S18 can be written in a form similar to Eq. 1. For spin configuration shown in Supplementary Fig. 5a, the Eq. S18 becomes:

$$P_a^2P_b^2 + P_a^2P_c^2 + P_b^2P_c^2 + 2\beta\mu_{\mathrm{Cr}}\mu_{\mathrm{Mn}}\Omega P_aP_bP_c = 0 \quad \text{(S19)}$$

The obtained Eq. S19 and Eq. 3 are fully consistent with Eq. 1 for $\delta = \pm 2\beta\mu_{\mathrm{Cr}}\mu_{\mathrm{Mn}}\Omega$, respectively. Form Eq. S19, the spin-induced **P** on the Roman surface is shown in

Supplementary Fig. 5c.

4. The Monte Carlo simulations under a small rotating B

To describe the external fields **B** dependent spin configurations, we write the following Hamiltonian of a local-moment Heisenberg model by including the AFM interlayer exchange interaction coupling J<0, cubic magnetic anisotropy constants K_1, K_2 and Zeeman energy, the total energy H is given by:

$$H = J\mathbf{S}_1 \cdot \mathbf{S}_2 + K_1 \sum_i (S_{i,x}^2 S_{i,y}^2 + S_{i,y}^2 S_{i,z}^2 + S_{i,z}^2 S_{i,x}^2) + K_2 \sum_i (S_{i,x}^2 S_{i,y}^2 S_{i,z}^2) - g\mu_B \mathbf{B} \cdot \sum_i \mathbf{S}_i \qquad \text{(S20)}$$

where, for the cubic lattice, we omit the higher order anisotropy terms and i = 1,2 for two antiparallelly aligned Cr spins. Given the easy axis to be [1,1,1], we could determine the relations between parameters:

$$K_2 < -9K_1 \text{ and } K_1 > 0, \text{ or, } 4K_2 < -9K_1 \text{ and } K_1 < 0 \qquad \text{(S21)}$$

By selecting tentative parameters satisfying the above conditions, K_1 = -4, K_2 = -2, J = 20, $g\mu_B B/S$ = 8, Monte Carlo simulations are run with small field rotating in the *ab*-plane as an example, using 30000 Monte Carlo steps per angle to equilibrate the structure and an additional 30000 steps to calculate the average magnetic properties. To be consistent with experimental data, we begin the simulations with T = 0.1 K and rotate the angle in equal steps, using the final state from the previous field as the initial state for the current field.

Under small magnetic field, the AFM configuration will cant slightly to induce a ferromagnetic component which is very small comparing to the saturation value while its AFM axis will also deviate from diagonal directions. The dominating change of **P** will mainly come from the deviation of AFM axis (Neel vector $\mathbf{S}_{Cr}$). As shown in Supplementary Fig. 7, the antiferromagnetic Neel vector $\mathbf{S}_{Cr} \propto (\mathbf{S_1} - \mathbf{S}_2)/2$ is tuned in

double period under **B**. The simulated data are fitted with sinusoidal functions in the forms:

$$S_{Cr}^{x} = 0.54 + 0.03\sin[2(\Phi - 48°)] \quad (S22)$$

$$S_{Cr}^{y} = 0.54 + 0.03\sin[2(\Phi - 131°)] \quad (S23)$$

$$S_{Cr}^{z} = 0.58 + 0.01\sin[2(\Phi + 4°)] \quad (S24)$$

where Φ is the angle of the external field in the *ab* plane. Then, the corresponding polarizations on the Roman surface have the following form:

$$P_x = 0.63 + 0.04\sin[2(\Phi + 40°)] \quad (S25)$$

$$P_y = 0.63 - 0.04\sin[2(\Phi + 51°)] \quad (S26)$$

$$P_z = 0.58 - 0.01\sin[2(\Phi + 5°)] \quad (S27)$$

Clearly, when the external field rotates 360°, the $\mathbf{S}_{Cr}$ and **P** draw a closed loop twice on the sphere and Roman surface, as shown in Supplementary Fig. 6a and b, respectively. When the small **B** is rotated along other planes, the situation would be similar.

Supplementary Table 1 | Typical two-dimensional topological manifolds (surface), their topological invariants, and their realization in physics. Realization of Roman surface in terms of multiferroicity and magnetoelectricity in TMCO and $LaMn_3Cr_4O_{12}$.

2D topological manifolds	Image	Orientability	Genus	Physical entity	Space
Sphere $\mathbf{S}^2$	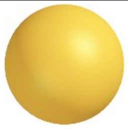	Orientable	0	Skyrmion[5,6], polar skyrmion[7], polar meron[8]	Real
Torus $\mathbf{T}^2$	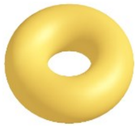		1	1st Brillouin zone	Reciprocal
Möbius strip	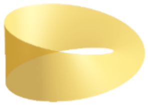	Non-orientable	1	Nanocrystal/DNA band[9,10], Möbius aromaticity in cycloalkene[11]	Real
Klein bottle	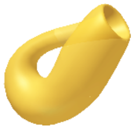		2	Energy landscape of cyclo-octane[12]	Phase
Roman surface $\mathbf{RP}^2$	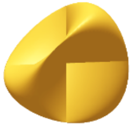		1	**Multiferroicity/ Magnetoelectricity (This Work)**	Phase

Supplementary Table 2 | Structure parameters of TMCO obtained from the Rietveld refinement at room temperature. The atomic position: Tb 2*a* (0, 0, 0), Mn 6*b* (0, 0.5, 0.5), Cr 8*c* (0.25, 0.25, 0.25), O 24*g* (*x*, *y*, 0). The bond valence sum (BVS) values (V_i) were calculating using the formula $V_i = \sum_j S_{ij}$ and $S_{ij} = \exp[(r_0 - r_{ij})/0.37]$. The parameters of $r_0(Mn^{3+})$ = 1.732 Å and $r_0(Cr^{3+})$ = 1.708 Å were used in calculations.

Parameters	$TbMn_3Cr_4O_{12}$
a (Å)	7.35288(1)
V (Å^3)	397.532(2)
O_x	0.3062(2)
O_y	0.1763(2)
U_{iso}(Tb) (100 × Å^2)	1.13(4)
U_{iso}(Mn) (100 × Å^2)	0.66(6)
U_{iso}(Cr) (100 × Å^2)	0.26(5)
U_{iso}(O) (100 × Å^2)	1.03(6)
Mn-O(× 4) (Å)	1.913(2)
Cr-O(× 6) (Å)	1.965(9)
BVS(Mn)	2.72
BVS(Cr)	2.99
R_{wp} (%)	2.41
R_p (%)	1.58

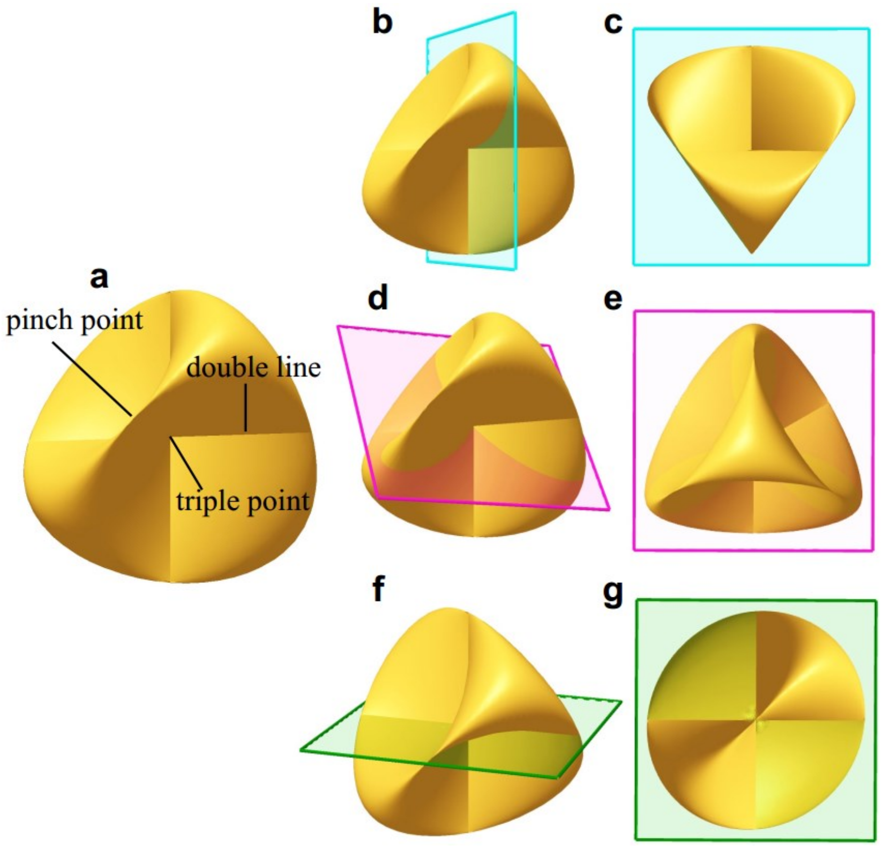

Supplementary Fig. 1 | Schematic diagram of Roman surface. a, The diagram of Roman surface with one triple point, six pinch points and three double lines. **b**, **d** and **f**, The different cross sections on Roman surface. Both cross sections in **b** and **d** pass through a double line and two extreme points on the surface. Another cross section in **f** passes through two double lines on the surface. **c**, **e** and **g**, The perspectives perpendicular to cross sections showing in **b**, **d** and **f**, respectively.

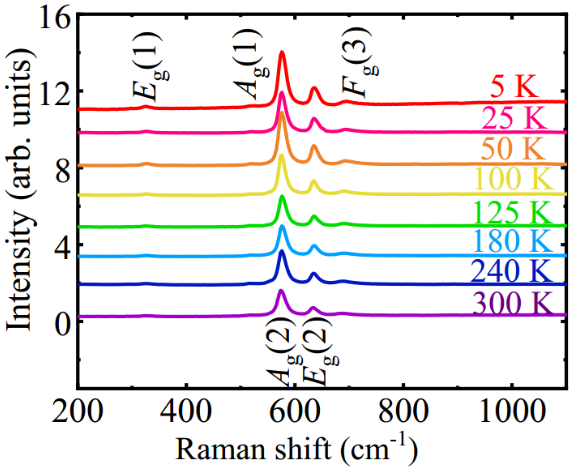

Supplementary Fig. 2 | Raman spectra measured for TMCO. Raman spectra measured at different temperatures where different Raman modes are also indicated. No structural phase transition is found to occur with temperature down to 5 K.

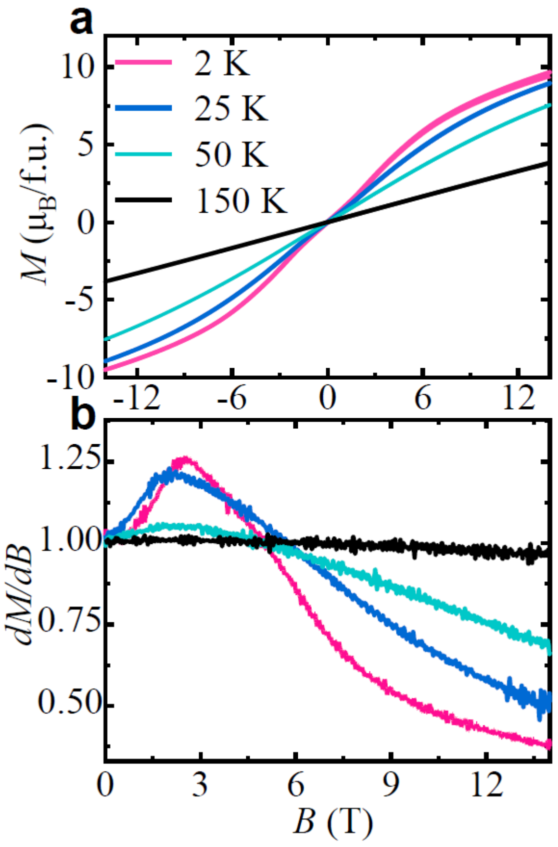

Supplementary Fig. 3 | Magnetic field dependent magnetization in TMCO. a, Isothermal magnetization curves measured at selected temperatures. **b**, Magnetic field dependence of the derivation of magnetization. The data are normalized by dividing the value obtained at 0 T. A metamagnetic transition is observed around 2.5 T below $T_{N1} \approx$ 136 K.

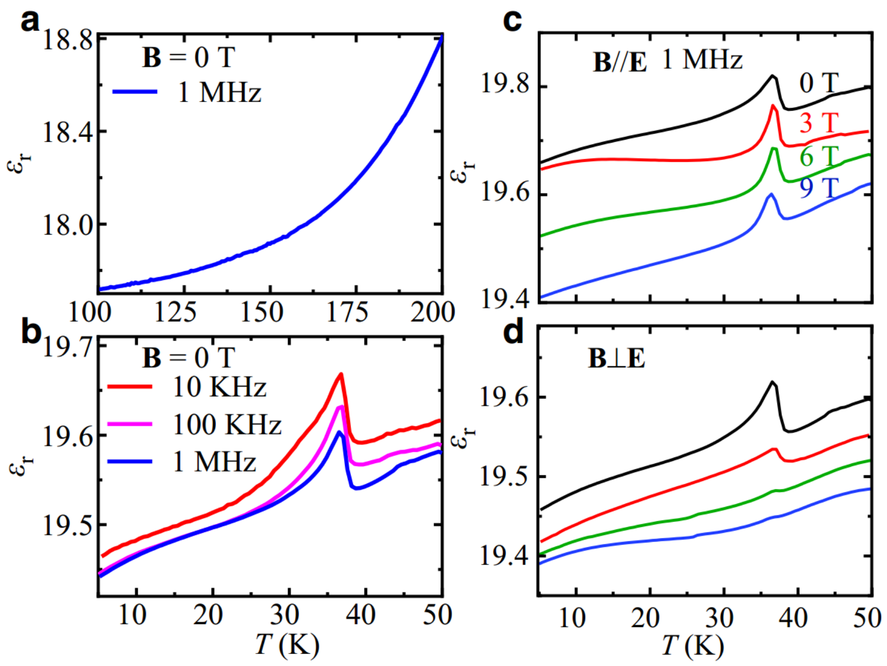

Supplementary Fig. 4 | Temperature dependence of dielectric constant ε_r for TMCO. a, Temperature dependent ε_r from 100 K to 200 K measured at 1 MHz under zero magnetic field, and the applied electric field E is about 11.7 kV/m. No dielectric anomaly is found to occur at T_{N1}. **b**, Temperature dependent ε_r from 5 K to 50 K measured at different frequencies under zero magnetic field. The frequency independent dielectric peak occurring at T_{N2} suggests a spin-induced ferroelectricity. **c**, **d**, Temperature dependence of ε_r measured at 1 MHz under different magnetic fields **B** applied in parallel and perpendicular to the electric field **E**, respectively. The data are shifted for clarity.

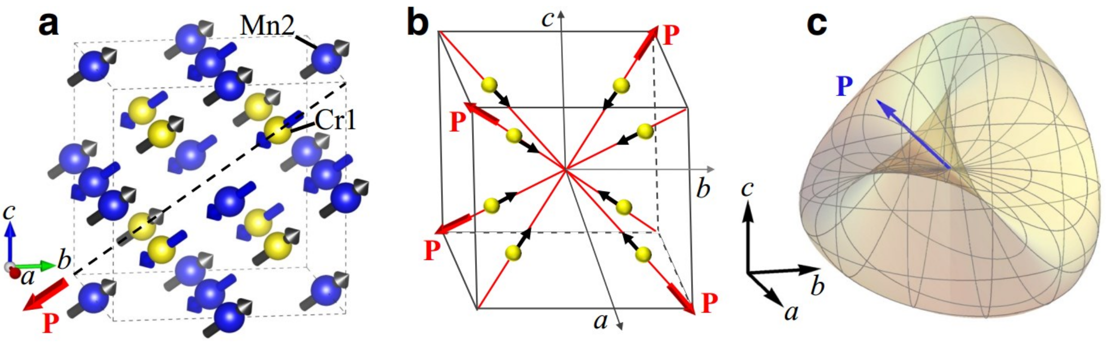

Supplementary Fig. 5 | Schematic diagram for the other magnetic and ferroelectric structures in *A*$Mn_3Cr_4O_{12}$. a, Spin configuration for the other magnetic structure in which Néel vector $\mathbf{S}_{Cr}$ is always antiparallel to $\mathbf{S}_{Mn}$ while they are parallel in Fig. 3a. This magnetic configuration is linked with that shown in Fig. 3a by the space inversion operation at the center and induces polarization with opposite direction. **b**, Distribution of **P** vectors along four out of eight diagonal directions with the spin configuration in Supplementary. Fig. 5a. **c**, The complete set of allowed spin-induced **P** vector in three dimensions by the magnetic structure in the Supplementary Fig. 5a.

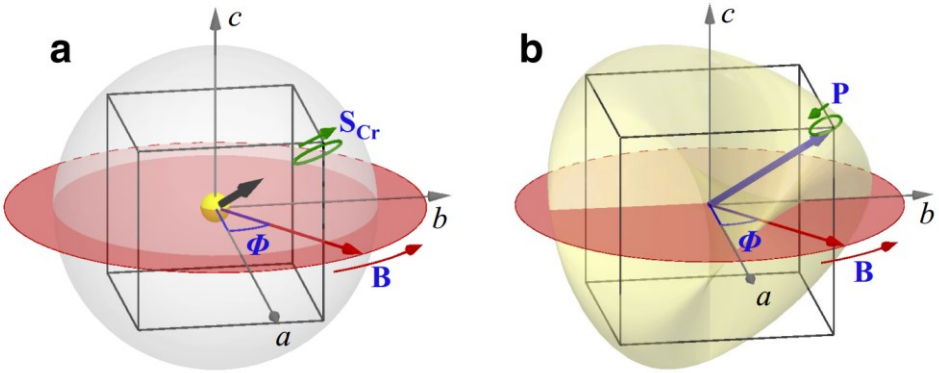

Supplementary Fig. 6 | Fields dependent loops on sphere of Néel vectors and the induced polarization trajectory on Roman surface. a, Néel vector rotation mode (green loop) effected by the rotating fields **B**. Φ is the angle of between *a*-axis and **B** in the *ab* plane while the shadow in red indicates the plane of rotation of **B**. **b**, Trajectory of **P** in green induced by loops in **a**. As the applied magnetic field rotates 360°, the $\mathbf{S}_{Cr}$ and **P** draw a closed loop twice on the sphere and Roman surface, respectively.

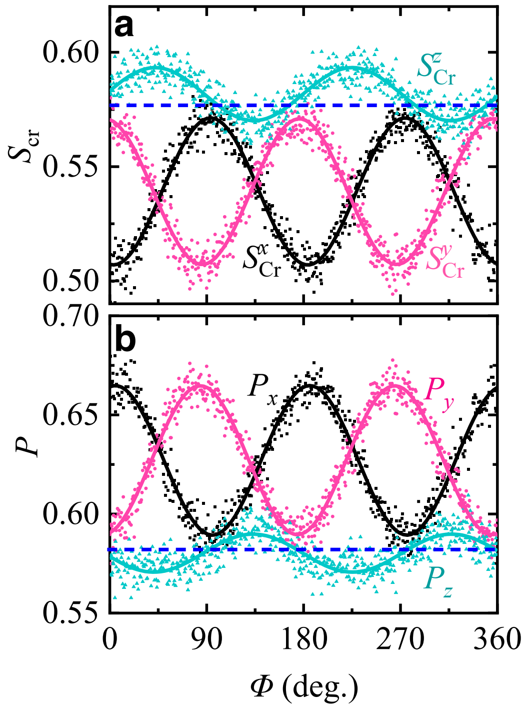

Supplementary Fig. 7 | Angle Φ dependent (a) $\mathbf{S}_{Cr}$ and (b) P. The dots are data from Monte Carlo simulations and the solid lines are fitted sinusoidal curves with double frequency. The Néel vector $\mathbf{S}_{Cr}$ and induced **P** are tuned in double period under **B**.

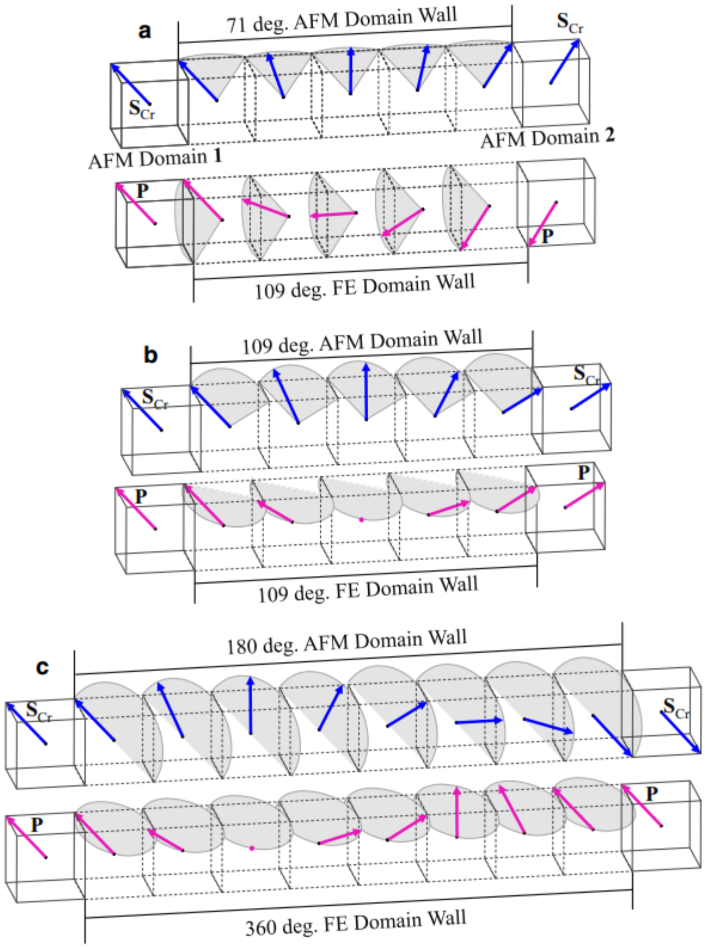

Supplementary Fig. 8 | Schematic diagram of three types of multiferroic domain walls with S_{Mn} parallel to S_{Cr} in each domain. (a) 71 deg., (b) 109 deg. and (c) 180 deg. AFM domain walls accompanied with corresponding ferroelectric (FE) domain walls by (a) 109 deg., (b) 109 deg. and (c) 360 deg., respectively. The solid black cells stand for AFM domains and corresponding ferroelectric domains. The dotted black cells are domain walls where shadows indicate rotation planes of Néel vector $\mathbf{S}_{Cr}$ and induced **P**. The continuously variation of AFM configuration is represented by the $\mathbf{S}_{Cr}$ in blue arrows. The induced **P** vectors by $\mathbf{S}_{Cr}$ inside ferroelectric domain wall are indicated by magenta arrows. In particular, the 180 deg. AFM/360 deg. ferroelectric domain wall would be topologically protected under external electric field.

5. References

1. Jia, C., Onoda, S., Nagaosa, N. & Han, J. H. Bond electronic polarization induced by spin. *Phys. Rev. B* **74**, 224444 (2006).
2. Murakawa, H., Onose, Y., Miyahara, S., Furukawa, N. & Tokura, Y. Ferroelectricity induced by spin-dependent metal-ligand hybridization in $Ba_2CoGe_2O_7$. *Phys. Rev. Lett.* **105**, 137202 (2010).
3. Feng, J. S. & Xiang, H. J. Anisotropic symmetric exchange as a new mechanism for multiferroicity. *Phys. Rev. B* **93**, 174416 (2016).
4. Chai, Y. S., Chun, S. H., Cong, J. Z. & Kim, K. H. Magnetoelectricity in multiferroic hexaferrites as understood by crystal symmetry analyses. *Phys. Rev. B* **98**, 104416 (2018).
5. Mühlbauer, S. et al. Skyrmion lattice in a chiral magnet. *Science* **323**, 915-919 (2009).
6. Yu, X. Z. et al. Real-space observation of a two-dimensional skyrmion crystal. *Nature* **465**, 901-904 (2010).
7. Das, S. et al. Observation of room-temperature polar skyrmions. *Nature* **568**, 368-372 (2019).
8. Wang, Y. J. et al. Polar meron lattice in strained oxide ferroelectrics. *Nat. Mater.* **19**, 881-886 (2020).
9. Tanda, S. et al. A Möbius strip of single crystals. *Nature* **417**, 397-398 (2002).
10. Han, D., Pal, S., Liu, Y. & Yan, H. Folding and cutting DNA into reconfigurable topological nanostructures. *Nat. Nanotechnol.* **5**, 712-717 (2010).

11. Ajami, D., Oeckler, O., Simon, A. & Herges, R. Synthesis of a Möbius aromatic hydrocarbon. *Nature* **426**, 819-821 (2003).

12. Martin, S., Thompson, A., Coutsias, E. A. & Watson, J.-P. Topology of cyclo-octane energy landscape. *J. Chem. Phys.* **132**, 234115 (2010).
